# Analysis of the association between racial inequities and edentulism in Brazil: a systematic review and meta-analysis

**DOI:** 10.1590/0102-311XEN210723

**Published:** 2025-11-10

**Authors:** Bianca Oliveira de Carvalho, Rodrigo Galo, Yure Gonçalves Gusmão, Maria Eliza da Consolação Soares

**Affiliations:** 1 Universidade Federal dos Vales de Jequitinhonha e Mucuri, Diamantina, Brasil.; 2 Faculdade de Odontologia de Ribeirão Preto, Universidade de São Paulo, Ribeirão Preto, Brasil.; 3 Universidade Federal de Juiz de Fora, Juiz de Fora, Brasil.

**Keywords:** Tooth Loss, Racial Groups, Ethnicity, Health Inequities, Perda de Dente, Grupos Raciais, Etnicidade, Desigualdades em Saúde, Pérdida de Diente, Grupos Raciales, Etnicidad, Inequidades en Salud

## Abstract

This study aimed to evaluate whether individuals who self-identify as black and/or mixed-race have a higher prevalence of tooth loss compared to white individuals in Brazil, using a systematic review and meta-analysis. Searches were conducted in the PubMed, Scopus, Web of Science, Virtual Health Library, Embase, and gray literature databases. Two independent reviewers performed the searches and article selection processes. The *Newcastle-Ottawa Scale* was used for observational cohort studies, and its modified version was used for cross-sectional studies. The I^2^ statistic assessed the heterogeneity of studies included in the meta-analyses. Of the 25 articles eligible for qualitative evaluation, 17 were included in the quantitative assessment. Sample sizes ranged from 101 to 18,718 individuals aged 11 to 74 years. Most studies compared white individuals to non-white individuals (black, mixed-race, Asian, and Indigenous people). In the comparison between white and non-white individuals, no differences were found concerning edentulism (OR = 0.86; 95%CI: 0.71; 1.06), absence of functional dentition (OR = 0.82; 95%CI: 0.33; 2.03), or mean number of missing teeth (MD = -0.21; 95%CI: -2.92; 2.49), but it was associated with tooth loss (OR = 1.40; 95%CI: 1.26; 1.55). When comparing black/mixed-race people to white individuals, tooth loss was higher among those who self-identified as black/mixed-race (OR = 1.41; 95%CI: 1.27; 1.57). This difference was also observed when comparing black/mixed-race individuals to other races/skin color (OR = 1.24; 95%CI: 1.15; 1.33). Overall, studies conducted in Brazil found that tooth loss was more prevalent among self-declared black and/or mixed-race individuals.

## Abstract (pt)

O objetivo deste estudo foi avaliar se indivíduos que se autoidentificam como pretos e/ou pardos apresentam maior prevalência de perda dentária em comparação a indivíduos brancos no Brasil por meio de uma revisão sistemática e metanálise. Foram consultadas as bases de dados PubMed, Scopus, Web of Science, Biblioteca Virtual em Saúe e Embase e literatura cinzenta. Dois revisores independentes realizaram as buscas e a seleção dos artigos. A *Escala de Newcastle-Ottawa* foi utilizada para estudos de coorte observacionais e sua versão modificada foi utilizada para estudos transversais. A estatística I^2^ foi utilizada para avaliar a heterogeneidade entre os estudos incluídos nas metanálises. Dos 25 artigos elegíveis para avaliação qualitativa, 17 foram submetidos à avaliação quantitativa. O tamanho da amostra variou de 101 a 18.718 indivíduos e idade variou de 11 e 74 anos. A maioria dos estudos comparou brancos com não brancos (pretos, pardos, asiáticos e indígenas). Na análise da comparação entre brancos e não brancos, não foram encontradas diferenças quanto ao edentulismo (OR = 0,86; IC95%: 0,71; 1,06), à ausência de dentição funcional (OR = 0,82; IC95%: 0,33; 2,03) ou ao número médio de dentes perdidos (DM = -0,21; IC95%: -2,92; 2,49), porém uma diferença significativa foi encontrada em relação à perda dentária (OR = 1,40; IC95%: 1,26; 1,55). Em comparação aos brancos, a perda dentária foi maior em indivíduos que se autodeclararam pretos/pardos (OR = 1,41; IC95%: 1,27; 1,57). Essa diferença também foi observada ao comparar pretos/pardos com outras raças/cores de pele (OR = 1,24; IC95%: 1,15; 1,33). Assim, em estudos realizados no Brasil, a perda dentária foi mais prevalente em indivíduos que se autodeclararam pretos e/ou pardos.

## Abstract (es)

El objetivo de este estudio fue evaluar si las personas que se identifican a sí mismas como negras y/o morenas tienen una mayor prevalencia de pérdida de dientes en comparación con las personas blancas en Brasil, mediante una revisión sistemática y un metaanálisis. Se consultaron las bases de datos PubMed, Scopus, Web of Science, Biblioteca Virtual en Salud, Embase y la literatura gris. Dos revisores independientes realizaron las búsquedas y la selección de artículos. Se utilizó la *Escala Newcastle-Ottawa* para los estudios de cohortes observacionales y su versión modificada para los estudios transversales. Se utilizó el índice I^2^ para evaluar la heterogeneidad de los estudios incluidos en los metaanálisis. De los 25 artículos aptos para la evaluación cualitativa, 17 formaron parte de la evaluación cuantitativa. El tamaño de la muestra osciló entre 101 y 18.718 personas de entre 11 y 74 años. La mayoría de los estudios compararon a personas blancas con personas no blancas (negras, morenas, asiáticas e indígenas). En el análisis de la comparación entre blancos y no blancos, no se encontraron diferencias en cuanto al edentulismo (OR = 0,86; IC95%: 0,71; 1,06), la ausencia de dentición funcional (OR = 0,82; IC95%: 0,33; 2,03), el número medio de dientes perdidos (MD = -0,21; IC95%: -2,92; 2,49); pero se encontró asociación con la pérdida de dientes (OR = 1,40; IC95%: 1,26; 1,55). Al comparar a las personas de raza negra/morenas con las de raza blanca, la pérdida de dientes fue mayor en las personas que se declararon de raza negra/morenas (OR = 1,41; IC95%: 1,27; 1,57). Esta diferencia también se observó al comparar a las personas de raza negra/morenas con otras razas/colores de piel (OR = 1,24; IC95%: 1,15; 1,33). Por lo tanto, en los estudios realizados en Brasil, la pérdida de dientes fue más frecuente en las personas que se declararon negras y/o morenas.

## Introduction

Racial inequity in health refers to the unfair distribution of health risks and resources ^1^. It can also be explained by structural racism, which encompasses the ways in which societies promote the maintenance of racial hierarchies ^2^. According to the most recent Brazilian demographic census ^3^, 55.9% of the population self-identifies as black or mixed-race. Despite certain advances, health inequities in this population persist significantly ^4^.

Unfavorable social trajectories are linked to poorer health outcomes, influencing indicators such as infant mortality, maternal mortality ratio, infectious diseases, chronic diseases, and health risk behaviors ^2^. Oral health also reflects these lived experiences, since individuals with low socioeconomic status, low schooling, and reduced participation in the labor market carry the marks of an unequal reality in oral health ^5^. Economic and social disadvantages, combined with inadequate access to health care, contribute to the persistence of health inequities ^5^ ^,^ ^6^ ^,^ ^7^.

Among the primary oral health issues, tooth loss stands out due to its high prevalence and its aesthetic, functional, and psychological impacts ^8^. Its effect on quality of life also includes reduced social acceptance and limited participation in the labor market ^9^. Caries are the main cause of tooth loss ^10^ ^,^ ^11^ ^,^ ^12^, followed by periodontal diseases and dental trauma ^10^. In addition to clinical causes, studies have shown that demographic and socioeconomic factors, health-related practices, and access to health services favor the occurrence of tooth loss ^11^. Considering these aspects, inequalities in oral health have been consistently reported through ethnic and racial disparities in different oral health indicators ^5^ ^,^ ^13^. An epidemiological study ^14^ suggested that black and/or mixed-race individuals have a higher prevalence of tooth loss than white individuals. Other two epidemiological studies ^15^ ^,^ ^16^ also shows that racial inequities in oral health in Brazil are evident across all indicators analyzed (cavities, tooth loss, pain, and need for prosthetics), with the black population (black and mixed-race) being more vulnerable than the white population. However, other studies did not observe such differences ^10^ ^,^ ^17^. In a recent systematic review ^18^, the authors investigated racial inequities considering all health conditions, including tooth loss; however, this review included studies conducted in other countries and did not perform meta-analyses.

Hence, it is essential to recognize the demands of specific groups and promote the creation of public policies aimed at reducing racial inequities and disparities in access to health services. Thus, identifying and analyzing the available evidence on racial inequality in oral health in Brazil can support the formulation of preventive strategies and the delivery of oral health care. Therefore, this systematic review and meta-analysis aimed to evaluate whether individuals who self-identify as black and/or mixed-race have a higher prevalence of tooth loss/edentulism compared to those who self-identify as white in Brazil.

## Material and methods

### Study design

This is a systematic literature review conducted in scientific databases, following the *Preferred Reporting Items for Systematic Reviews and Meta-Analyses* (PRISMA) guidelines ^19^. The study protocol was registered in the International Prospective Register of Systematic Reviews (PROSPERO; CRD42022337441).

The research question guiding this review was: “Do individuals (P) who self-identify as black and/or mixed-race (E) have a difference in tooth loss (localized or total) (O) compared to individuals who are white in Brazil (C)?”. Accordingly, the PECO strategy (Population, Exposure, Comparison, Outcome) was applied: P corresponds to the study population; E to the exposure factor; C compares exposed and unexposed individuals, and O refers to the outcome.

### Eligibility criteria

The eligibility criteria for this review were as follows: studies conducted in Brazil that addressed tooth loss and edentulism in children, adults, and older people belonging to black, mixed-race, or white racial groups; and observational studies (longitudinal, cross-sectional, or case-control) that assessed tooth loss/edentulism via clinical examination and race/skin color.

Studies that evaluated tooth loss and/or edentulism via self-report and those not conducted in Brazil were excluded.

### Search strategy and databases

The main sources used to identify publications are electronic databases, manual searches and gray literature searches. Thus, searches were conducted in the following databases: PubMed, Scopus, Web of Science, Virtual Health Library and Embase. The gray literature on Google Scholar, the Brazilian Digital Library of Theses and Dissertations, and the reference lists of included studies were also consulted. No restrictions were applied regarding language and date of publication. The last search was conducted on June 26, 2022.

The search strategy was developed individually for each database, combining the following terms and their variations: tooth loss, healthcare disparities, racial groups, race, ethnicity, and Brazil (Box 1).

**Box 1:** Search strategy in different databases.

DATABASE	SEARCH STRATEGY
MEDLINE (via PubMed)	(((Racial Groups) OR (race) OR (Ethnicity) OR (racial stock) OR (Blacks) OR (Negro) OR (Ethnic Group) OR (negroes) OR (Negroid Race) OR (Ethnic and Racial Minorities) OR (Ethnic Minority) OR (Racial Minority) OR (race factor) OR (racial factor) OR (African Continental Ancestry Group) OR (racial inequalities) OR (White; European Continental Ancestry Group) OR (Caucasoid Race) OR (Caucasian Race)) AND ((Dental Caries) OR (Dental Decay) OR (Dentin, Carious) OR (Periodontal Diseases) OR (Parodontosis) OR (Parodontoses) OR (Pyorrhea Alveolaris) OR (Tooth Loss) OR (Mouth, Edentulous) OR (Mouth, Toothless) OR (Jaw, Edentulous, Partially) OR (DMF Indices) OR (Decayed, Missing, and Filled Teeth) OR (Dental Prosthesis) OR (Removable Partial Denture) OR (Denture complete) OR (edentulism) OR (DMF Index) OR (Jaw, Edentulous)) AND ((brazil) OR (Brazilian)))
Web of Science (via CAPES Periodicals)	(Racial Groups OR race OR Ethnicity OR racial stock OR Blacks OR Negro OR Ethnic Group OR negroes OR Negroid Race OR Ethnic and Racial Minorities OR Ethnic Minority OR Racial Minority OR race factor OR racial factor OR African Continental Ancestry Group OR racial inequalities OR White European Continental Ancestry Group OR Caucasoid Race OR Caucasian Race) AND (Dental Caries OR Dental Decay OR Dentin, Carious OR Periodontal Diseases OR Parodontosis OR Parodontoses OR Pyorrhea Alveolaris OR Tooth Loss OR Mouth, Edentulous OR Mouth, Toothless OR Jaw, Edentulous, Partially OR DMF Indices OR Decayed, Missing, and Filled Teeth OR Dental Prosthesis OR Removable Partial Denture OR Denture complete OR edentulism OR DMF Index OR Jaw, Edentulous) AND (brazil OR Brazilian)
Virtual Health Library	(((Racial Groups) OR (race) OR (Ethnicity) OR (racial stock) OR (Blacks) OR (Negro) OR (Ethnic Group) OR (negroes) OR (Negroid Race) OR (Ethnic and Racial Minorities) OR (Ethnic Minority) OR (Racial Minority) OR (race factor) OR (racial factor) OR (African Continental Ancestry Group) OR (racial inequalities) OR (White; European Continental Ancestry Group) OR (Caucasoid Race) OR (Caucasian Race)) AND ((Dental Caries) OR (Dental Decay) OR (Dentin, Carious) OR (Periodontal Diseases) OR (Parodontosis) OR (Parodontoses) OR (Pyorrhea Alveolaris) OR (Tooth Loss) OR (Mouth, Edentulous) OR (Mouth, Toothless) OR (Jaw, Edentulous, Partially) OR (DMF Indices) OR (Decayed, Missing, and Filled Teeth) OR (Dental Prosthesis) OR (Removable Partial Denture) OR (Denture complete) OR (edentulism) OR (DMF Index) OR (Jaw, Edentulous)) AND ((brazil) OR (Brazilian)))
Scopus (via CAPES Periodicals)	(((Racial Groups) OR (race) OR (Ethnicity) OR (racial stock) OR (Blacks) OR (Negro) OR (Ethnic Group) OR (negroes) OR (Negroid Race) OR (Ethnic and Racial Minorities) OR (Ethnic Minority) OR (Racial Minority) OR (race factor) OR (racial factor) OR (African Continental Ancestry Group) OR (racial inequalities) OR (White; European Continental Ancestry Group) OR (Caucasoid Race) OR (Caucasian Race)) AND ((Dental Caries) OR (Dental Decay) OR (Dentin, Carious) OR (Periodontal Diseases) OR (Parodontosis) OR (Parodontoses) OR (Pyorrhea Alveolaris) OR (Tooth Loss) OR (Mouth, Edentulous) OR (Mouth, Toothless) OR (Jaw, Edentulous, Partially) OR (DMF Indices) OR (Decayed, Missing, and Filled Teeth) OR (Dental Prosthesis) OR (Removable Partial Denture) OR (Denture complete) OR (edentulism) OR (DMF Index) OR (Jaw, Edentulous)) AND ((brazil) OR (Brazilian)))
Embase (via CAPES Periodicals)	(‘ancestry group’ OR race OR ethnicity OR ‘Black person’ OR ‘ethnic group’ OR ‘racial disparity’ OR Caucasian) AND (‘dental caries’ OR ‘dentin carious’ OR ‘periodontal disease’ OR periodontosis OR ‘pyorrhea alveolaris’ OR ‘tooth loss’ OR edentulism OR ‘edentulous jaw’ OR ‘DMF index’ OR ‘DMFT index’ OR ‘tooth prosthesis’ OR ‘complete denture’) AND (Brazil OR Brazilian)
Google Scholar	((Tooth loss) AND (Healthcare Disparities) AND (Racial Groups) AND (Race) AND (Ethnicity) AND (Brazil))
Brazilian Digital Library of Theses and Dissertations	(((Racial Groups) OR (race) OR (Ethnicity) OR (racial stock) OR (Blacks) OR (Negro) OR (Ethnic Group) OR (negroes) OR (Negroid Race) OR (Ethnic and Racial Minorities) OR (Ethnic Minority) OR (Racial Minority) OR (race factor) OR (racial factor) OR (African Continental Ancestry Group) OR (racial inequalities) OR (White; European Continental Ancestry Group) OR (Caucasoid Race) OR (Caucasian Race)) AND ((Dental Caries) OR (Dental Decay) OR (Dentin, Carious) OR (Periodontal Diseases) OR (Parodontosis) OR (Parodontoses) OR (Pyorrhea Alveolaris) OR (Tooth Loss) OR (Mouth, Edentulous) OR (Mouth, Toothless) OR (Jaw, Edentulous, Partially) OR (DMF Indices) OR (Decayed, Missing, and Filled Teeth) OR (Dental Prosthesis) OR (Removable Partial Denture) OR (Denture complete) OR (edentulism) OR (DMF Index) OR (Jaw, Edentulous)) AND ((brazil) OR (Brazilian)))

### Study selection and data extraction

After searching each database, all retrieved references were exported to EndNote 20 (http://www.endnote.com/). Reviewer calibration was assessed before the selection process using Cohen’s kappa test on 20% of the articles, yielding a satisfactory agreement (κ > 0.8).

The first stage of selection involved removing duplicates. Subsequently, two independent reviewers screened the titles and abstracts of the retrieved references. Full texts were retrieved for studies whose titles or abstracts lacked sufficient information for a decision on inclusion/exclusion. Next, the reviewers evaluated the full-text articles and included those meeting the eligibility criteria. In cases of disagreements between the two reviewers, a third researcher was consulted.

The following information from each included study was independently evaluated: author(s), year of publication, city/region, publication language, study design, method of tooth loss assessment, skin color classification, sample size (participants and/or tooth loss cases), gender, age, race/skin color, type of tooth loss, findings related to tooth loss, previous edentulism, use and need for prostheses, dental caries, periodontal disease, and socioeconomic status. The authors were contacted by e-mail to clarify any unclear information in the manuscripts.

### Risk of bias assessment

The *Newcastle-Ottawa Scale* for observational studies was used to assess the quality of cohort studies ^20^. This scale evaluates eight items grouped into three categories. In the selection category, each item (representativeness of the exposed cohort, selection of the unexposed cohort, determination of exposure, and confirmation that the outcome was absent at the beginning of the study) received up to one point, totaling four points. In the comparability category (one item), two points could be assigned, and in the results category (outcome analysis, adequate follow-up period, and completeness of cohort follow-up), each item receives one point. Thus, each study could receive a maximum of nine points.

The modified *Newcastle-Ottawa Scale* for cross-sectional studies was applied to assess the risk of bias of eligible studies ^20^, analyzing six items with a maximum total score of seven points ^21^. In the selection category, each item (sample representativeness, non-respondents, exposure measurement) received up to one point, totaling three points. The comparability and result categories each consisted of a single item, with up to two points allocated across them.

Two independent reviewers assessed the risk of bias and disagreements were resolved by discussion with a third reviewer. Cohort studies were classified as high risk of bias (0-3 points), moderate risk (4-6 points), or low risk (≥ 7 points) ^22^. Cross-sectional studies were classified as high risk (0-3 points), moderate risk (4-6 points), or low risk (7 points).

### Data synthesis and statistical analysis

For data synthesis, a narrative summary of the results from the included studies was performed, structured around the outcome (tooth loss/edentulism) and characteristics of the target population. Sufficiently homogeneous studies regarding outcome measures and result presentation were analyzed with meta-analysis using the RevMan 5.5 software (https://revman.cochrane.org/info). Heterogeneity was assessed using the I^2^ statistic and considered high when I^2^ > 50%. Thus, a random-effects model was used when I^2^ > 50%, and a fixed-effect model was used when I^2^ ≤ 50%. Odds ratios (OR) for dichotomous outcomes - such as tooth loss prevalence, edentulism, and absence of functional dentition - were calculated with 95% confidence intervals (95%CI). Continuous effect size measures were analyzed using mean differences. The comparison groups in the meta-analyses were white versus non-white, black/mixed-race versus white, and black/mixed-race versus other races/ethnicities. Forest plots were generated for each analysis.

## Results

### Study selection 

**Figure 1:**
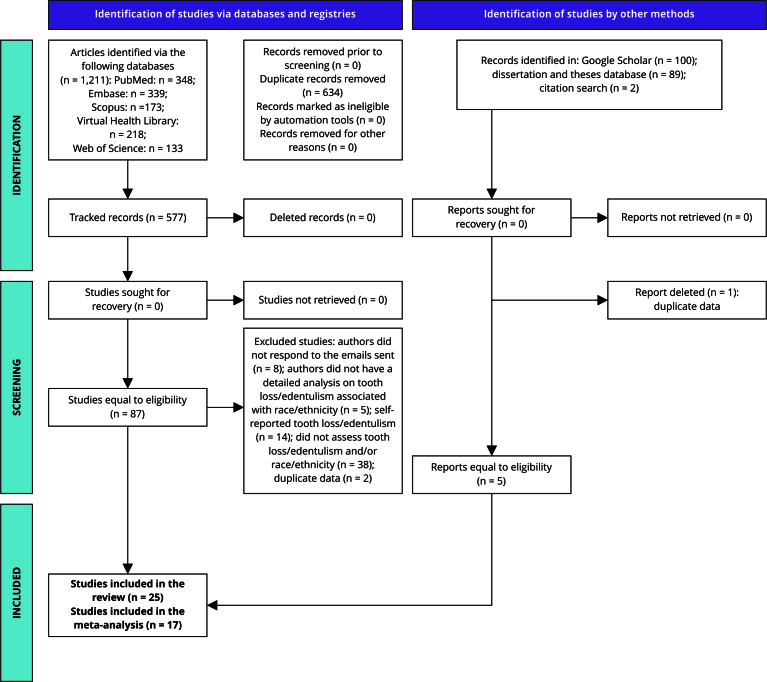
PRISMA flowchart of the systematic review selection process.

A total of 1,211 articles were retrieved. After removing 634 duplicates, 577 studies remained for titles and abstract screening. Of these, 87 articles were selected for full-text review, of which 67 were excluded for not meeting the eligibility criteria. In the gray literature search, 191 studies were retrieved; after analysis, 186 were excluded, including one duplicate study, leaving only five articles eligible. Thus, 25 articles met the eligibility criteria for this review, of which 17 were included in the quantitative evaluation (meta-analysis) (Figure 1).

### Characteristics of the included studies

Box 2 shows the characteristics of eligible studies. Of the 25 eligible studies, 24 were cross-sectional and one was a cohort. Articles were published between 2003 and 2021. Sample sizes ranged from 101 to 18,718 individuals, whose ages ranged from 11 to 74 years or older. Regarding geographic location, 11 studies were conducted in the Southeast Region ^12^ ^,^ ^17^ ^,^ ^23^ ^,^ ^24^ ^,^ ^25^ ^,^ ^26^ ^,^ ^27^ ^,^ ^28^ ^,^ ^29^ ^,^ ^30^ ^,^ ^31^, four in the South Region ^32^ ^,^ ^33^ ^,^ ^34^ ^,^ ^35^, one study in the Northeast ^36^, and the remaining nine ^5^ ^,^ ^8^ ^,^ ^37^ ^,^ ^38^ ^,^ ^39^ ^,^ ^40^ ^,^ ^41^ ^,^ ^42^ ^,^ ^43^ were nationwide epidemiological surveys. Race/ethnicity self-declaration followed the Brazilian Institute of Geography and Statistics (IBGE, acronym in Portuguese) classification. The included studies used oral clinical examination; the decayed, missing, and filled teeth (DMFT) index for permanent teeth; and the DMFT index for primary teeth to assess outcomes. Some studies did not evaluate tooth loss, edentulism, mean number of missing teeth, absence of functional dentition, or prosthesis use or need as primary outcomes ^5^ ^,^ ^23^ ^,^ ^24^ ^,^ ^25^ ^,^ ^26^ ^,^ ^27^ ^,^ ^29^ ^,^ ^31^ ^,^ ^33^ ^,^ ^34^ ^,^ ^35^ ^,^ ^36^ ^,^ ^38^ ^,^ ^43^. However, as they reported relevant data on these variables, they were included in this review.

**Box 2:** General characteristics of the included studies.

STUDY (YEAR)	LOCATION	SAMPLE ORIGIN	STUDY DESIGN	SAMPLE SIZE	AGE GROUP	DENTITION	VARIABLES STUDIED	MAIN RESULTS
Antoniazzi et al. ^32^ (2021)	Santa Maria (Rio Grande do Sul State)	Studying in a public institution	Cross-sectional	106 cocaine and crack users and 106 controls matched for age, sex, and smoking status	13 to 46 years old	Permanent	Dental caries: DMFT Tooth loss: presence or absence. Periodontal disease: presence or absence Skin color: (white and non-white)	Multivariate analysis revealed that tooth loss was significantly higher among non-white individuals, those over 24 years old, and those with severe caries (p-value < 0.001)
Antunes et al. ^23^ (2003)	São Paulo State	Population-based survey	Cross-sectional	18,718 oral examination records	11 and 12 years old	Mixed	Dental caries: DMFT Care Index Tooth loss: proportion of missing teeth Ethnicity: white (European ancestry); black (Afro-descendant)	White children had higher caries rates in permanent teeth than black children The difference in caries prevalence between black and white children was reduced in cities with a better socioeconomic status profile Caucasian children also had a better profile of cared-for teeth, with lower numbers of missing and untreated decayed teeth, higher rates of attendance, lower averages of teeth with preventive and restorative treatment needs, and higher proportions of children without dental treatment
Azevedo et al. ^37^ (2017)	Brazil	Population-based survey	Cross-sectional	7,496	65 to 74 years old	Permanent	Edentulism: use or need for dentures Race/color: white, black, mixed-race, yellow, Indigenous	After adjusting for confounding variables, females were associated with greater use of prostheses, as well as those with five to seven years of schooling, compared to those with fewer years of education. black and mixed-race individuals had lower use of prostheses compared to white individuals Prevalence of prosthesis use (p < 0.001 ) Prevalence of need for prosthesis (p = 0.008)
Carvalho ^24^ (2012)	Reginópolis (São Paulo State)	Population-based survey	Cross-sectional	101 adolescents	11 to 13 years old 14 to 16 years old	Permanent	Dental caries: DMFT Tooth loss: component “P” of DMFT Periodontal disease: CPI Quality of life: OHIP-14 Significant caries index Dental Care Index (caries) Ethnicity: white and non-white (yellow, mixed-race, black)	The carious component showed a similar mean regarding age and sex However, the mean was higher than that of non-white adolescents living in the rural area of the municipality The prevalence of gingival bleeding and dental calculus was also observed in adolescents aged 11 to 13 years who were white and lived in urban areas (p-value = 0.297)
Colaço et al. ^33^ (2020)	Cruz Alta (Rio Grande do Sul State)	Household surveys	Cross-sectional	287 older people	The median age was 69 years old	Permanent	Tooth loss: clinical examination Quality of life: OHIP-14 Ethnicity/race: white and non-white (black, mixed-race, yellow, Indigenous)	Divorced individuals and those who reported not using dental floss had 77% and 54%, respectively, the highest prevalence ratio of having a more significant impact on OHRQoL Older adults who do not need dental prostheses showed less effect on OHRQoL (p < 0.01)
Drummond ^38^ (2016)	Brazil	Population-based survey	Cross-sectional	2003 (n = 16,833) and 2010 (n = 5,367)	15 to 19 years old	Permanent	Tooth decay: DMFT Tooth loss: DMFT component “P” (0 = no tooth loss, ≥ 1 tooth loss) Race/ethnicity: white, people of African descent, people of East-Asian descent, mixed-race, Indigenous descent	White individuals had fewer untreated decayed teeth, and mixed-race individuals had more missing teeth A hierarchical conceptual model analysis confirmed the association between untreated caries, missing and restored teeth, and race/ethnicity The adjusted odds ratio confirmed that, compared to white individuals, Mixed-race individuals were 1.52 times more likely to have missing teeth, and black and mixed-race groups were 0.72 and 0.89 times less likely to have restored teeth
Moreira et al. ^39^ (2010)	Brazil	Population-based survey	Cross-sectional	13,431 subjects	35 to 44 years old	Permanent	Tooth loss: clinical examination measured on a scale of 0 to 32 Ethnicity: caucasian and other	When adjusted for the variables studied, ethnicity did not affect tooth loss However, having nine or more years of schooling was associated with protection against tooth loss Not having been to the dentist and not having been to the dentist in the last three years represented increases of 33.5% and 21.3%, respectively, in the risk of tooth loss (p < 0.05)
Gaio et al. ^34^ (2012)	Porto Alegre (Rio Grande do Sul State)	Population-based survey	Cross-sectional	217 subjects	60 years old or older	Permanent	Edentulism: defined as the complete absence of all teeth Dental caries and periodontal disease: clinical examination Race: white and non-white	Edentulism was significantly more likely to occur in older individuals, women, white individuals, those with low socioeconomic status, and smokers (p = 0.01) Race-associated periodontitis and race-associated mean periodontal attachment loss were not statistically significant, p = 0.92 and p = 0.44, respectively
Guiotoku et al. ^5^ (2012)	Brazil	Population-based survey	Cross-sectional	12,811 adults	35 to 44 years old	Mixed	Caries experience: DMFT Tooth loss: number of missing teeth Anterior edentulism: yes or no Dental pain experience: yes or no Need for prosthesis: 0 = no need; 1 = need for partial denture; 2 = need for full dentures Race/color: white, black, and mixed-race	Correlations were found between oral health outcomes and indicators related to human development profile, average family income, and income inequality by the brazilian state for the black group Mean DMFT associated with race/ethnicity (p < 0.01) Mean tooth loss (number of teeth) (p < 0.01) Number of anterior edentulism (p < 0.01) Need for partial dentures (p < 0.01) Need for total dentures (p < 0.01)
Gushi et al. ^25^ (2005)	São Paulo State	Population-based survey	Cross-sectional	1,825 adolescents	15 to 19 years old	Permanent	Dental caries: DMFT Tooth loss: Component “P” of DMFT Ethnicity: white and non-white	There were differences in components of the DMFT index: the non-white group had a higher percentage of decayed (p = 0.000) and missing (p = 0.000) teeth At the same time, white individuals had a higher rate of restored teeth (p = 0.000)
Lopez et al. ^40^ (2010)	Brazil	Population-based survey	Cross-sectional	13,431 subjects	35 and 44 years old	Permanent	Dental caries: DMFT Periodontal disease: CPI Tooth loss: absence and presence Ethnic group: Asian, white, Indigenous, black	Regarding ethnicity, results showed a loss of six teeth across all ethnicities examined However, white individuals had fewer missing teeth than the other ethnic groups
Martins et al. ^31^ (2007)	Brazil	Population-based survey	Cross-sectional	5,319 seniors	65 to 74 years old	Permanent	Dental caries: DMFT Edentulous: use and necessity of prosthesis Periodontal disease: CPI Race: white and non-white	Among the edentulous, the use was higher among those with a higher level of education and those who reported pain sensitivity and lower among older people identified as non-white and those who did not receive information on oral health There was no association with the distribution of edentulous subjects according to race (p = 0.091)
Mendes et al. ^26^ (2012)	Montes Claros (Minas Gerais State)	Studying in a public institution	Cross-sectional	200 older people	60 years old or older	Permanent	Dental caries: DMFT (absence and present) Edentulism: absence of teeth Periodontal disease: CPI (absence and present) Ethnicity: white and non-white	The study showed no statistical difference considering dental caries, periodontal disease, edentulism, and lesions in the oral mucosa associated with ethnicity Ethnicity and dental caries (p= 0.674) Ethnicity and periodontal disease (p= 0.509) Ethnicity and edentulism (p = 0.085)
Nogueira et al. ^27^ (2019)	São Paulo State	Population-based survey	Cross-sectional	6,051 adults	35 to 44 years old	Permanent	Dental caries: DMFT Missing teeth: dichotomized by median in < 4 > 4 Ethnicity: white and non-white	Income up to BRL 1,500, schooling up to 8 years, low and medium social capital, and women were more likely to have missing teeth The results also showed that income of up to BRL 1,500, schooling up to 8 years and non-white ethnicities were more likely to have decayed teeth
Peres et al. ^8^ (2013)	Brazil	Population-based survey	Cross-sectional	5,445 (15 and 19 years old) 9,779 (35 and 44 years old) 7,619 (65 and 74 years old)	15 to 74 years old	Permanent	Dental caries: DMFT Prevalence of individuals without functional dentition: presence of < 21 natural teeth Edentulism: total loss of all teeth Skin color: white, mixed-race, black, yellow, Indigenous	Among adolescents, women, mixed-race, and black individuals, those with lower income and schooling had a higher prevalence of losses Absence of functional dentition occurred in approximately 1/4 of adults, being higher among women, black, and mixed-race groups and those with lower income and schooling Higher prevalences of edentulism in older people were observed in women and those with lower income and education Prevalence of tooth loss and ethnicity (p = 0.039) Prevalence of no functional dentition and ethnicity (p = 0.019) Prevalence of edentulous and ethnicity (p = 0.662)
Prado ^42^ (2015)	Brazil	Population-based survey	Cross-sectional	9,779 7,619	35 to 44 years old 65 to 74 years old	Permanent	Dental caries: DMFT Periodontal disease: CPI Ethnicity: white, black, mixed-race, other	All ethnic groups, both adults and older adults, had a high prevalence of impacts compared to white ethnicity Adjusted prevalence ratio for tooth loss and race in older people (p < 0.001) Adjusted prevalence ratio for tooth loss and race in adults (p < 0.001)
Rihs et al. ^29^ (2009)	São Paulo (São Paulo State)	Population-based survey	Cross-sectional	1,192 older people	65 to 74 years old	Permanent	Dental caries: DMFT Tooth loss: component “P” of DMFT Race: Caucasian and non-Caucasian	There was no association between tooth absence and race (p < 0.32); however, regarding race, DMFT was the only variable that showed differences between groups, with higher DMFT for Caucasians (p < 0.02)
Rihs et al. ^28^ (2009)	São Paulo (São Paulo State)	Population-based survey	Cross-sectional	1,159 teachers and school workers	35 to 44 years old	Permanent	Dental caries: DMFT Tooth loss: “P” component of DMFT Ethnicity: white and non-white	The white group had more caries-free individuals and a higher mean number of teeth than the non-white group (p < 0.05) The DMFT index was higher for men and non-white individuals, and the missing teeth component was higher among men, older people, non-white individuals, and those living in regions without water fluoridation
Sachetti et al. ^35^ (2020)	Cruz Alta and Veranópolis (Rio Grande do Sul State)	Population-based survey	Cross-sectional	569 subjects	60 years old or older	Permanent	Tooth loss was categorized into edentulism (no teeth at all) and non-edentulism (one or more teeth) Race/skin color: white and non-white (black, mixed-race, yellow, Indigenous)	Cruz Alta: mean tooth loss in white individuals was 19.57, and 29.6% of the sample were edentulous Of non-white individuals, 19.96 had a mean tooth loss, and 30.8% of were edentulous Veranópolis: mean tooth loss in white individuals was 20.69, and 48.8% were edentulous Of non-white individuals, mean tooth loss was 20.93, and 46.7% were edentulous Older adults without access to a dentist in the last 12 months had a higher prevalence ratio (p = 0.006) of concern about dental appearance than those with access to dental care Older people with teeth had a 219% higher prevalence ratio for concern with dental appearance compared to edentulous individuals
Silva Júnior et al. ^12^ (2019)	Piracicaba (São Paulo State)	Population-based survey	Cohort	143 adults	20 to 64 years old	Permanent	Dental caries: DMFT Periodontal disease: CPI Tooth loss: 0 (no missing) and 1 (missing ≥ 1 tooth in 4 years) Skin color: White and Non-white (black, mixed-race, yellow, or Indigenous)	The incidence of tooth loss due to ethnicity was not statistically significant (p = 0.801) Among risk factors for tooth loss, the following were reasons for seeking dental services due to pain: decayed teeth
Sanchez ^30^ (2018)	São Paulo (São Paulo State)	Population-based survey	Cross-sectional	5,951 older people	65 years old or older	Permanent	Functional dentition: ≥ to 21 teeth < 21 teeth Ethnicity/race: Caucasian and non-Caucasian	Regarding ethnicity, 12.65% of Caucasian and 9.97% of non-Caucasian had functional dentition, compared to 87.35% of Caucasian and 90.03% of non-Caucasian who did not have functional dentition There was no association with the frequency of the presence of functional dentition according to ethnicity/race (p < 0.1)
Brizolara ^17^ (2017)	São Paulo (São Paulo State)	Population-based survey	Cross-sectional	373 adults in 2010 and 308 in 2015	35 to 44 years old	Permanent	Edentulism and tooth loss: absent and present Functional dentition loss: < 20 functional teeth Dental caries: DMFT Periodontal disease: CPI Ethnic group: White and non-white (yellow or Indigenous, mixed-race or black)	Edentulism and ethnic group: no statistical association was observed in 2010 (p = 0.5323) and 2015 (p = 0.4736) Tooth loss and ethnic group: no statistical association between 2010 (p = 0.1961) and 2015 (p = 0.2068) Loss of functional dentition and ethnic group: no statistical association between 2010 (p = 0.8114) and 2015 (p = 0.6364)
Martins et al. ^41^ (2009)	Brazil	Population-based survey	Cross-sectional	241 subjects	35 to 44 years old	Permanent	Tooth loss and edentulism: component “P” of DMFT Ethnic group consisting of two categories: white and yellow; and black and mixed-race	Among variables at the individual level, monthly income of less than 170 dollars, household density of more than one individual per room, being black or mixed-race, no family member having insurance, and medical care were associated with the loss of 12 teeth or more in individuals over 40 years of age Risk factors for tooth loss: being black or mixed-race (p = 0.032)
Landim et al. ^36^ (2013)	Fortaleza (Ceará State)	Household surveys	Cross-sectional	141 subjects	Over 35 years old	Permanent	Dental caries: DMFT Edentulism: use or need for dentures Race/ethnicity: yellow, black, mixed-race, Indigenous	Economic class and race/ethnicity did not statistically show their relationship with the use and need for upper and lower dentures, having been related only to the higher use of lower dentures among mixed-race individuals In the black population, 118 people, which represents the most significant portion of the sample, had a lower average number of decayed teeth than that of white and Indigenous groups Need for upper prosthesis (p = 0.496) Need for lower prosthesis (p = 0.069)
Meira ^43^ (2016)	Brazil	Population-based survey	Cross-sectional	7,208	12 years old	Mixed	Dental caries: DMFT (presence or absence) Gingivitis: CPI (presence or absence) Tooth loss: component “P) of DMFT (P ≥ 1) Race/ethnicity: white, mixed-race, black, yellow, and Indigenous	Dental caries, tooth loss, and gingivitis predominated in mixed-race male children, with low family income, and among those with five years or less of schooling Mixed-race children with lower family income and fewer household assets were more likely to have a higher number of oral clinical conditions, suggesting that the number of oral medical conditions is related to social inequities

CPI: Community Periodontal Index; DMFT: Decay, Missing and Filled Teeth Index; OHIP-14: *Oral Health Impact* Profile; OHRQoL: oral health related quality of life.

In two studies ^26^ ^,^ ^41^, edentulism was evaluated; two studies ^17^ ^,^ ^30^ assessed the absence of functional dentition; eight ^24^ ^,^ ^25^ ^,^ ^28^ ^,^ ^29^ ^,^ ^33^ ^,^ ^35^ ^,^ ^39^ ^,^ ^40^ evaluated the mean number of missing teeth; five ^12^ ^,^ ^32^ ^,^ ^38^ ^,^ ^42^ ^,^ ^43^ assessed the loss of at least one tooth; two studies ^36^ ^,^ ^37^ evaluated the use and need for prostheses, and seven ^5^ ^,^ ^8^ ^,^ ^23^ ^,^ ^27^ ^,^ ^31^ ^,^ ^34^ evaluated more than one outcome (edentulism, loss of at least one tooth, mean tooth loss, and/or absence of functional dentition). In 22 studies, prevalence of tooth loss was evaluated in permanent dentition ^8^ ^,^ ^12^ ^,^ ^17^ ^,^ ^24^ ^,^ ^25^ ^,^ ^26^ ^,^ ^27^ ^,^ ^28^ ^,^ ^29^ ^,^ ^30^ ^,^ ^31^ ^,^ ^32^ ^,^ ^33^ ^,^ ^34^ ^,^ ^35^ ^,^ ^36^ ^,^ ^37^ ^,^ ^38^ ^,^ ^39^ ^,^ ^40^ ^,^ ^41^ ^,^ ^42^, while three assessed prevalence of tooth loss in mixed dentition ^5^ ^,^ ^23^ ^,^ ^43^. No studies evaluated tooth loss or indication for extraction in primary dentition.

### Risk of bias assessment

Of the 24 cross-sectional studies included, nine were classified as having high methodological quality and 15 as having moderate quality (Box 3). The main limitation identified among them was related to response rates. The sole cohort study was classified as having high methodological quality (Boxes 4 and 5).

**Box 3:** Risk assessment of included studies.

STUDIES	SELECTION	COMPARABILITY	OUTCOME	RESULT	CLASSIFICATION
Cohort					
1	★ ★ ★ ★	★ ★ ★	★	8★	High quality
Cross-sectional					
9	★ ★ ★	★ ★	★ ★	7★	High quality
15	★ ★	★ ★	★ ★	6★	Intermediate quality

**Box 4:** Assessment of the quality of studies according to the *Newcastle-Ottawa Scale* (adapted for cross-sectional studies).

STUDY (YEAR)	SELECTION			COMPARABILITY		RESULT		
	REPRESENTATIVENESS OF THE EXPOSED	NON-RESPONDENTS	DETERMINATION OF EXPOSURE (RISK FACTOR)	COMPARABILITY OF GROUPS BASED ON DESIGN OR ANALYSIS		EVALUATION OF RESULTS	STATISTICAL TEST	SCORE
Antoniazzi et al. ^32^ (2021)	★	★	★	★	★	★	★	7
Antunes et al. ^23^ (2003)	★		★	★	★	★	★	6
Azevedo et al. ^37^ (2017)	★		★	★	★	★	★	6
Carvalho ^24^ (2012)	★	★	★	★	★	★	★	7
Colaço et al. ^33^ (2020)	★	★	★	★	★	★	★	7
Moreira et al. ^39^ (2010)	★		★	★	★	★	★	6
Drummond ^38^ (2016)	★		★	★	★	★	★	6
Gaio et al. ^34^ (2012)	★		★	★	★	★	★	6
Guiotoku et al. ^5^ (2012)	★		★	★	★	★	★	6
Gushi et al. ^25^ (2005)	★	★	★	★	★	★	★	7
Lopez et al. ^40^ (2010)	★		★	★	★	★	★	6
Martins et al. ^31^ (2007)	★		★	★	★	★	★	6
Mendes et al. ^26^ (2012)	★	★	★	★	★	★	★	7
Nogueira et al. ^27^ (2019)	★		★	★	★	★	★	6
Rihs, et al. ^29^ (2009)	★		★	★	★	★	★	6
Rihs et al. ^28^ (2009)	★	★	★	★	★	★	★	7
Sachetti et al. ^35^ (2020)	★	★	★	★	★	★	★	7
Peres et al. ^8^ (2013)	★		★	★	★	★	★	6
Sanchez ^30^ (2018)	★		★	★	★	★	★	6
Brizolara ^17^ (2017)	★		★	★	★	★	★	6
Martins et al. ^41^ (2009)	★	★	★	★	★	★	★	7
Prado ^42^ (2015)	★		★	★	★	★	★	6
Landim et al. ^36^ (2013)	★	★	★	★	★	★	★	7
Meira ^43^ (2016)	★		★	★	★	★	★	6

**Box 5:** Quality assessment of cohort studies according to the *Newcastle-Ottawa Scale*.

STUDY (YEAR)	SELECTION			COMPARABILITY				RESULT		
	REPRESENTATIVENESS OF THE EXPOSED COHORT	SELECTION OF THE UNEXPOSED COHORT	DETERMINATION OF EXPOSURE	DEMONSTRATION THAT THE DEVELOPMENT OF INTEREST WAS NOT PRESENTED AT THE BEGINNING OF THE STUDY	COMPARABILITY OF COHORTS BASED ON DESIGN OR ANALYSIS		RESULT EVALUATION	FOLLOW-UP WAS LONG ENOUGH FOR THE RESULTS TO OCCUR		SUITABILITY OF MONITORING OF COHORTS	SCORE
Silva Júnior et al. ^12^ (2019)	★	★	★	★	★	★	★	★		8

### Synthesis of results and meta-analysis

**Figure 2:**
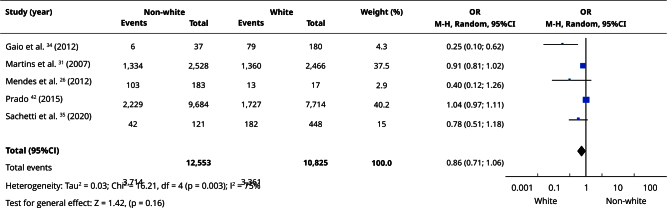
Forest plot of the comparison of non-white versus white individuals for edentulism.

**Figure 3:**
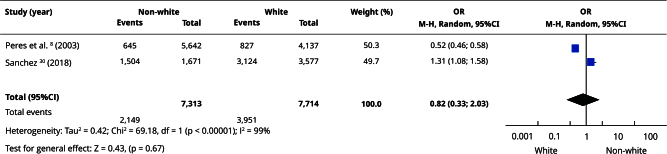
Forest plot of the comparison of non-white versus white individuals for the absence of functional dentition.

**Figure 4:**
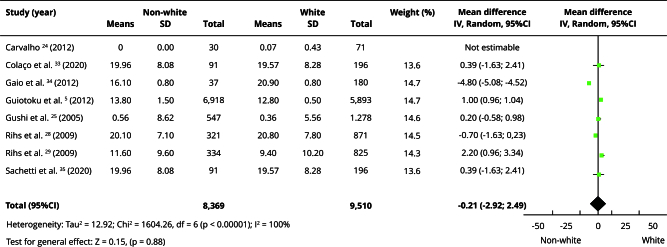
Forest plot of the comparison between non-white and white individuals for average lost teeth.

**Figure 5:**
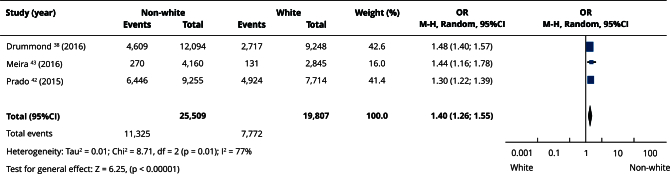
Forest plot of the comparison between non-white and white individuals for tooth loss.

**Figure 6:**
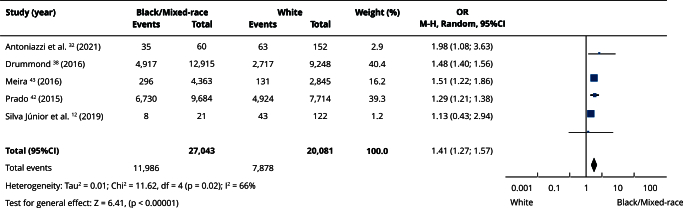
Forest chart of the comparison between black/mixed-race versus white individuals for tooth loss.

**Figure 7:**
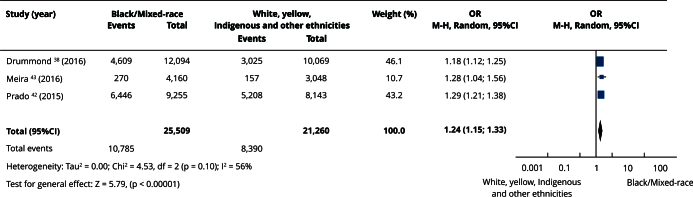
Forest plot of the comparison between black/mixed-race versus white, yellow, Indigenous and other ethnicities individuals for tooth loss.

In the qualitative evaluation (n = 25), 17 studies provided sufficient data and presented some degree of homogeneity, making it possible to include them in the meta-analyses. Most studies categorized participants as white or non-white, and analyses using this classification had the highest number of articles. Comparisons between white and non-white individuals showed no statistically significant differences in the prevalence of edentulism (OR = 0.86; 95%CI: 0.71; 1.06) (Figure 2) or absence of functional dentition (OR = 0.82; 95%CI: 0.33; 2.03) (Figure 3). Similarly, there was no difference in the mean number of missing teeth between white and non-white individuals (mean difference - MD = -0.21; 95%CI: -2.92; 2.49) (Figure 4). When considering the presence of at least one missing tooth, the frequency was higher among non-white individuals (OR = 1.40; 95%CI: 1.26; 1.55) (Figure 5). Only three studies were included in the meta-analysis comparing blacks/mixed-race individuals with white individuals. It was observed that tooth loss was more prevalent in the black/mixed-race group (OR = 1.41; 95%CI: 1.27; 1.57) (Figure 6). A similar analysis comparing black/mixed-race individuals with other racial groups also showed a statistical difference (OR = 1.24; 95%CI: 1.15; 1.33) (Figure 7). For the last two comparisons (black/mixed-race versus white and black/mixed-race versus other races), only the tooth loss outcome (at least one missing tooth) was included in the analyses due to differences in the dichotomization of race/skin color and in the way the results were presented in the primary studies.

95%CI: 95% confidence interval; M-H: Mantel-Haenszel method; OR: odds ratio.

95%CI: 95% confidence interval; M-H: Mantel-Haenszel method; OR: odds ratio.

95%CI: 95% confidence interval; IV: inverse variance; SD: standard deviation.

95%CI: 95% confidence interval; M-H: Mantel-Haenszel method; OR: odds ratio.

95%CI: 95% confidence interval; M-H: Mantel-Haenszel method; OR: odds ratio.

95%CI: 95% confidence interval; M-H: Mantel-Haenszel method; OR: odds ratio.

## Discussion

The meta-analyses results indicated that, among the variables studied, tooth loss was more prevalent in self-declared black/mixed-race individuals compared to other racial groups. Additionally, when comparing white and non-white individuals, tooth loss was higher in the latter group.

Tooth loss is a well-recognized marker of social inequality in several societies ^30^. According to Sanchez ^30^, low monthly family income and schooling levels can predispose to tooth loss. This study results suggest that race may also be associated with a higher prevalence of tooth loss. In the United States, studies have reported that black individuals are more likely to experience tooth loss compared to white individuals, even after adjusting for socioeconomic indicators such as income and education ^44^. A recent systematic review on racial inequities in oral health showed that black populations had a higher likelihood of tooth loss compared to white individuals ^18^.

In the meta-analyses of this review, no difference was observed between white and non-white groups regarding number of missing teeth. However, Drummond ^38^ showed that among racial groups, white individuals had fewer untreated decayed teeth, whereas mixed-race individuals had a higher number of missing teeth. Conversely, in other investigations, race/skin color lost significance when adjusted for other confounding variables ^12^ ^,^ ^28^ ^,^ ^34^ ^,^ ^39^. Notably, most studies assessing the number of missing teeth dichotomized skin color/race into whites and non-whites. This categorization may limit the detection of specific differences, particularly between black and mixed-race populations.

The association between the absence of functional dentition and skin color/race was investigated in three studies. In one of them ^8^, black and mixed-race individuals had a higher prevalence of lack of functional dentition than white individuals. However, the other two studies did not demonstrate such an association ^17^ ^,^ ^30^. Brizolara ^17^ states that differences in the definition and calculation criteria for functional dentition may limit methodological comparisons and affect the interpretation of outcomes. Regarding skin color/race, Peres et al. ^8^ followed the IBGE classification (white, yellow, mixed-race, black, and Indigenous), Sanchez ^30^ classified individuals as Caucasian and non-Caucasian, and Brizolara ^17^ grouped them into white and non-white categories.

Regarding edentulism, most included studies did not show an association with race/ethnicity ^8^ ^,^ ^17^ ^,^ ^26^ ^,^ ^28^ ^,^ ^42^. According to the authors, ethnicity as a risk marker in certain populations may be influenced by confounding factors. In a study ^45^ assessing individuals’ perceptions of edentulism, most participants attributed its occurrence to difficulties in accessing treatment. The authors highlight persistent barriers to specialized dental care within the Brazilian Unified National Health System (SUS, acronym in Portuguese), especially for endodontic treatments ^45^. Consequently, tooth extraction often becomes the only viable option in advanced caries cases, particularly among lower-income groups ^45^. A recent systematic review ^46^ found that access to dental services in Brazil remains unequal, being less frequent among people with lower education and income levels, as well as those who live in rural areas. Notably, no analyses to date have specifically addressed the relationship between race and endodontic treatment, indicating the need for further research on this topic.

Only two studies evaluated the use and need for prostheses, which precluded conducting a meta-analysis. Azevedo et al. ^37^ reported that black and mixed-race individuals had a lower prevalence of prosthesis use compared to white individuals, with no significant difference in prosthesis need. According to the authors ^37^, these results indicate disadvantages and exclusions in accessing more complex and costly services, such as prosthetic rehabilitation, among black and mixed-race populations. In contrast, Ladim et al. ^36^ found no association between race/ethnicity and the overall use or need for upper and lower prostheses; however, an association was observed between race and the use of lower prostheses.

In the meta-analyses, it was impossible to consider subgroups based on participants’ age, mainly due to the limited number of investigations involving children. However, studies conducted with children found that white children had a better oral health profile, with fewer decayed, missing, and untreated teeth, higher rates of dental care, and a lower need for restorative treatment ^23^ ^,^ ^43^. It is worth noting that, in the evaluated studies, the race/ethnicity variable for children aged 11 and over was based on self-classification, in which individuals identify themselves within specific race or ethnicity categories ^23^ ^,^ ^24^ ^,^ ^32^ ^,^ ^43^. In contrast, studies using hetero-identification - in which a person’s race or ethnicity is assigned by researchers, interviewers, or health professional rather than by the individual themselves - may introduce bias by tending to categorize individuals as white, leading to an underestimation of the black population ^47^.

Studies ^48^ ^,^ ^49^ suggest that racism is a fundamental cause of health disparities. It is understood as a structuring system that generates behaviors, practices, beliefs, and prejudices underlying avoidable and unjust inequalities based on race or ethnicity ^50^. This system encompasses several flexible resources that benefit white individuals across psychological, structural, individual, and social levels, reflecting socio-historical processes such as slavery. Therefore, the relationship between race and health inequalities permeates socioeconomic status and includes power dynamics, social prestige, neighborhood effects, stigmatization, and discrimination, all of which contribute to significant racial disparities in overall health and oral health ^48^ ^,^ ^51^ ^,^ ^52^. Additionally, institutional racism - a system of racial inequality present within public or private companies, including academic institutions - has been increasingly discussed ^53^. In the context of professional training, dental practitioners often exhibit behaviors that suggest limited reflection on racial issues, resulting in discrimination against specific population groups ^54^. Previous research in Recife (Pernambuco State), Northeast Brazil ^55^, demonstrated that, under equal clinical conditions, dentists recommended tooth extraction more frequently for black patients than for white patients, indicating that race may influence clinical decisions regarding the extraction or retention of decayed teeth. Conversely, a cross-sectional study conducted in the United States ^56^ found no association between racial discrimination and dental service use among adults after adjustments. Addressing stigmatization requires thorough and strategic efforts, especially regarding educational interventions aimed at transforming social beliefs and attitudes. As for discrimination, legislative and judicial actions are needed ^57^.

Health inequalities are influenced by different socioeconomic conditions ^1^. Brazil is a large country with natural, social, and economic differences in its territory. According to Peres et al. ^8^, individuals residing in the capitals and rural areas of the North and Northeast experience greater tooth loss than those in the South and Southeast. This disparity is attributed to differences in the coverage of preventive measures against tooth loss, such as water fluoridation, which is concentrated in the South and Southeast. Moreover, the use of and access to oral health services are known to be lower in economically disadvantaged regions ^58^. However, in the same study by Peres et al. ^8^, ethnicity lost its association with tooth loss after adjusting for socioeconomic variables, suggesting that social and economic conditions may have a greater influence than race in this context. Guiotoku et al. ^5^ found that most states with the lowest Human Development Index (HDI) and average family income were in the North and Northeast of Brazil. These states also presented the poorest oral health indicators for the black population. In this review, regional analysis was not feasible due to the limited number of studies and methodological differences between them.

The 2003 Brazilian National Oral Health Policy aimed to improve access to and increase the use of dental services within SUS, resulting in significant advances. Peres et al. ^8^ conducted a comparative analysis of tooth loss using data from Brazil’s two most recent major epidemiological surveys. The prevalence of tooth loss in adolescents and the absence of functional dentition in adults were higher among black and mixed-race individuals, while edentulism did not vary according to ethnicity. Although this study demonstrates a significant reduction in tooth loss in the Brazilian population, social and regional inequalities persist, suggesting that, in addition to universal measures, more vulnerable populations should receive priority care ^8^. While this policy was established in 2003, *Law n. 14,572* ^59^ was only recently enacted, formally recognizing the policy within SUS as a state law rather than merely a guideline supported by ordinances of the Brazilian Ministry of Health. Therefore, this legal framework is expected to promote the expansion and enhancement of specialized care with the implementation of Dental Specialty Centers (CEO, acronym in Portuguese) and Regional Dental Prosthesis Laboratories. Additionally, it aims to facilitate and increase the population’s access to free preventive dental treatment via SUS.

Some considerations should be given to the analyses in this review. The high heterogeneity found across all analyses is significant and may stem from methodological issues, sample sizes, geographic regions within Brazil, or years when the surveys were conducted. To address this limitation, random-effects models were used to achieve greater accuracy. Another important aspect is the lack of standardized criteria for classifying variables related to tooth loss and race/ethnicity. Most included studies classify individuals as white or non-white, which may have limited the ability to detect associations specific to black and mixed-race individuals. Such analysis was only possible for the tooth loss variable and included a limited number of articles. This limitation should be considered in future studies, since there are strong suggestions of racial inequities in oral health issues ^5^ ^,^ ^38^. It should be noted that multiple studies from the same database (national surveys) were included in this review; however, only those evaluating different age groups and outcomes were maintained. Despite the limitations, this is the first systematic review with meta-analysis to investigate tooth loss/edentulism across racial groups in Brazil. Furthermore, an extensive search was conducted in databases without restrictions on language or publication year, and most included studies had large sample sizes.

This systematic review found that most included studies used samples derived from population-based surveys and cross-sectional epidemiological designs. In population-based studies with adequate randomization, the selection path is minimized; however, this factor should be carefully considered, as some studies did not specify participant selection methods. The selection perspective occurs when the sample is not representative of the target population, potentially skewing results. The information perspective may also be present, in which interviewers consciously or unconsciously influence participants’ responses. The confounding perspective occurs when the relationship between exposure and outcome is distorted by a third factor associated with exposure and development, such as socioeconomic status ^60^. These potential perspectives should be considered when interpreting the results of this review and when designing future studies.

Regarding the methodological aspects, the included studies used different terms to classify self-declared race/ethnicity and tooth loss. Some studies used the IBGE-recommended categories; while most dichotomized variables into white and non-white, Caucasian and non-Caucasian, or used classifications such as Asian, white, Indigenous, and black. In most studies, data collection followed the IBGE standardized categories and later grouped participants into white or non-white categories. When analyzing the data, it is evident that authors often redefined or grouped racial categories according to their research objectives. While this practice can facilitate statistical analyses and comparisons, it may also obscure relevant nuances of racial inequality. The classification of individuals as white or non-white has been criticized in the scientific literature - especially within social sciences, public health, and epidemiology - for erasing identities, homogenizing diverse groups, and perpetuating racial inequalities ^61^. It is important to acknowledge that this review retained such classifications because many original studies reported their results accordingly. However, future research should address this issue by adopting a more accurate approach aligned with best practices.

It well established that the classification, measurement, and reporting of tooth loss in studies vary according to the age group investigated ^8^. Evidence suggests that, among adolescents, it is preferable to measure the prevalence of tooth loss rather than the number of teeth affected, since the disease has become relatively rare in this age group. For adults and older people, the absence of functional dentition and edentulism have been proposed as appropriate indicators. However, even for criteria like the lack of functional dentition, definitions vary across studies - for instance, some classify individuals with fewer than 21 natural teeth as lacking functional dentition, while others use a threshold of fewer than 20 natural teeth. Future studies should address the need to standardize the criteria for calculating the number of functional teeth, thereby facilitating comparisons across research findings and the classification of race/ethnicity. The use of different classification methods in the included studies led to methodological inconsistencies that restricted the performance of subgroup analyses by factors such as age, region, and other outcomes. We recognize that stratifying individuals into subgroups could better reflect the role of social inequality - determined in this study by ethnicity - in oral health outcomes.

Based on the evidence presented, the presence of racial inequities in oral health is suggested, as a higher prevalence of tooth loss was observed among black and/or mixed-race individuals compared to other racial groups. Considering the high prevalence of tooth loss in Brazil, mainly among individuals classified as black and/or mixed-race, it is necessary to adopt measures and strategies aimed at addressing this inequity. The engagement of researchers, civil society, policymakers, health professionals, and other social agents is essential to ensure that the findings of this and similar studies guarantee the improvement of the quality of life of these population. This study compiles evidence that may guide actions aimed at enhancing oral health among black and mixed-race individuals. Notably, the variables edentulism, mean number of missing teeth, and absence of functional dentition showed no significant differences. However, it was not possible to isolate black/mixed-race groups in the analysis, as most studies classified them as white and non-white.

## Conclusion

Tooth loss was more prevalent among self-declared black and/or mixed-race individuals compared to other racial/ethnicity groups. The included studies had moderate to high methodological quality. No association was found when comparing white and non-white individuals for edentulism, absence of functional dentition, or mean number of missing teeth. These conclusions should be interpreted with caution due to variations in the dichotomization of race/ethnicity, differences in the presentation of results in the primary studies, the small number of studies included in the meta-analyses, and the high heterogeneity among them.

## Data Availability

The sources of information used in the study are indicated in the body of the article.
